# Developing personas for nutritional self-management in nasopharyngeal carcinoma patients: a qualitative study based on the Fogg Behavior Model

**DOI:** 10.3389/fnut.2026.1920095

**Published:** 2026-09-01

**Authors:** Xuejiao Lei, Chunyu Wang, Jiayi Du, Suting Song, Yanchen Liu, Yao Liu

**Affiliations:** 1 Department of Radiotherapy, Chongqing University Cancer Hospital, Chongqing, China; 2 School of Medicine, Chongqing University, Chongqing, China; 3 Department of Hematologic Oncology, Chongqing University Cancer Hospital, Chongqing, China

**Keywords:** Fogg Behavior Model, nasopharyngeal carcinoma, nutritional self-management, patient persona, qualitative study

## Abstract

**Purpose:**

Malnutrition is common throughout the course of nasopharyngeal carcinoma and requires long-term self-management. Current standardized nutritional management models struggle to meet the diverse needs of patients. This study aimed to identify the behavioral characteristics and influencing factors of nutritional self-management among patients with nasopharyngeal carcinoma and to develop user personas.

**Methods:**

This study was conducted from July to September 2025 in a cancer hospital in Chongqing, China, and patients were recruited using purposive sampling. Data were collected through semi-structured interviews. Directed content analysis was conducted in NVivo 15, with themes organized inductively under the Fogg Behavior Model framework (motivation, ability, prompt). The process sequentially involved marking meaning units, extracting factual labels, and constructing dimension models. Personas were developed by categorizing patients based on these dimensions.

**Results:**

A total of 21 patients were enrolled, and a label framework spanning five dimensions was developed, including motivation, ability, prompt, psychological state, and family support. Five typical behavioral personas were identified: proactive managers, symptom-driven active managers, survival-driven passive copers, cognition-behavior disconnected individuals, and family-led passive compliers.

**Conclusion:**

Nutritional self-management behaviors in patients with nasopharyngeal carcinoma exhibit significant heterogeneity. Precise, personalized interventions can be provided based on patient personas. In the future, a nutritional management system could be developed based on personas, enabling dynamic updates to these personas to enhance the accuracy of interventions.

## 1 Introduction

Nasopharyngeal carcinoma (NPC) most commonly arises in the pharyngeal recesses of the roof and lateral walls of the nasopharynx. It is one of the most common malignant tumors in otolaryngology (1). Approximately 50% of new cases worldwide are concentrated in China, particularly in the southern regions (2). Currently, comprehensive treatment centered on radiotherapy can achieve a 5-year survival rate of 80% (3). However, patients' nutritional status generally deteriorates during treatment. Studies have shown that the prevalence of malnutrition among nasopharyngeal carcinoma patients before treatment ranges from 8.7% to 10.3%, while the proportion of patients experiencing weight loss of ≥5% during treatment is as high as 53.6% to 70.2% (4, 5). In addition to the energy expenditure caused by the tumor itself, radiotherapy damages the mucous membranes of the mouth, nose, and pharynx, leading to nutrition impact symptoms (NIS) such as oral mucositis, dry mouth, dysphagia, and loss of taste (6). Gastrointestinal reactions caused by concurrent chemotherapy, including nausea, vomiting, and anorexia, further exacerbate the risk of malnutrition (7). On the one hand, malnutrition suppresses immune function, reduces radiosensitivity, and increases treatment inaccuracies, significantly impairing treatment outcomes and prognosis (8, 9). On the other hand, symptoms such as fatigue and weakness caused by malnutrition reduce patients' quality of life, hinder their return to work, and impose a heavy economic burden on families and society (10, 11). Therefore, providing appropriate nutritional support is crucial during the treatment of nasopharyngeal carcinoma patients.

Effective nutritional management can improve nasopharyngeal cancer patients' attitudes and behaviors toward healthy eating, thereby optimizing their nutritional status and enhancing treatment tolerance and quality of life (12, 13). Xiao et al. (14) developed a nurse-led educational intervention program based on the Medical Research Council (MRC) framework to help patients with nasopharyngeal cancer cope with nutrition-related symptoms. Ji et al. (15) utilized the FOCUS-PDCA model to manage the nutritional status of nasopharyngeal carcinoma patients undergoing radiotherapy. However, existing studies primarily rely on evidence-based approaches to construct healthcare-provider-led nutritional management protocols, focusing mainly on evaluation dimensions, the scientific rigor and universality of educational content and intervention measures, resulting in relatively homogeneous management strategies (16, 17). Patients are typically passive recipients of treatment and management, but there are widespread individual differences in symptoms, nutritional motivation, cognition, knowledge, and dietary preferences (14, 18). These factors may further influence their self-directed nutritional management behaviors and willingness to take an active role in their care. Despite the growing emphasis on personalized nutrition, studies specifically addressing NPC patients' nutrition practices and individualized management remain limited, warranting further research.

Previous studies have mostly used single variables or scales for categorical analysis, but they have not delved deeply enough into the underlying causes of these differences. In contrast, User personas can generate various thematic dimensions and tags by integrating and analyzing patient text data, thereby creating more accurate, representative personas that facilitate the development of personalized nutrition management (19). Furthermore, these user personas can be integrated into online health information platforms and continuously updated in future versions, thereby providing more precise medical services (20). In recent years, user personas have been effectively applied in the healthcare sector (19, 20). However, research on constructing user personas for nutritional self-management in nasopharyngeal cancer remains limited. The Fogg Behavior Model (FBM), a classic theory in behavioral design, posits that behavior depends on three core elements: motivation, ability, and prompt (21). These elements correspond to the driving forces behind behavior, the skills and resources required to perform the behavior, and the timing and context in which the behavior is initiated. This model has been widely applied in the field of chronic disease self-management. It can elucidate the mechanisms underlying target behaviors and their influencing factors, thereby providing important theoretical support for developing intervention strategies and increasing the prevalence of health-promoting behaviors (22, 23). The FBM aligns closely with the objectives of this study and provides a basis for constructing an interview outline for nasopharyngeal cancer patients and delineating thematic dimensions.

In summary, this qualitative study, grounded in the FBM, used user profiling to integrate multi-source patient data systematically. This approach enabled the identification of representative nutritional self-management behaviors and population characteristics, revealing key differences across the three FBM components. The resulting user personas provide a foundation for developing tailored nutritional support strategies.

## 2 Methods

### 2.1 Study design

This study employed a qualitative descriptive research design based on the FBM. The reporting of the study procedures followed the recommendations outlined in the Consolidated Criteria for Reporting Qualitative Research (COREQ) checklist (24).

### 2.2 Participants

This study adopted purposive sampling to recruit patients with nasopharyngeal carcinoma from a Grade A tertiary cancer hospital in Chongqing, China, from July to September 2025. Maximum variation sampling was applied according to differences in age, gender, education level, family support, treatment stage, and nutritional status. Inclusion criteria: (a) Pathological diagnosis of nasopharyngeal carcinoma; (b) Age ≥ 18 years; (c) No cognitive or language impairment as determined by clinical assessment; (d) Informed consent and voluntary participation. Exclusion criteria: Presence of other serious diseases affecting the heart, brain, lungs, or other organs that significantly compromise physical health. The sample size was determined based on the principle of data saturation; interviews were terminated when no new themes emerged.

### 2.3 Interview guide

The initial interview guide was drafted based on the Fogg Behavior Model, focusing on the three elements (motivation, ability, prompt) that generate nutritional self-management behavior in nasopharyngeal carcinoma patients. It also examined patients' psychological experiences and family support for nutritional self-management. The initial interview outline was then refined through consultations with two oncology nursing experts and pilot interviews with two participants, as shown in Table 1.

**Table 1:** Interview guide.

Key interview questions	
1	Do you engage in dietary and nutritional management? Why or why not? (Motivation)
2	What specific abilities or resources do you have related to nutrition management? (Ability)
3	What channels do you use to learn nutritional knowledge? (Ability)
4	Which knowledge has been most helpful for your actual dietary adjustments? (Ability)
5	What conditions encourage you to follow a prescribed nutrition plan? (Prompt)
6	What factors or prompts lead to changes in your behavior? (Prompt)
7	What role do family members play in your nutrition management? (Motivation/Ability/Prompt)
8	How do you feel overall during the process of self-managing your nutrition? (Motivation)
9	What are the main barriers you currently face in self-managing your nutrition? (Ability)

### 2.4 Data collection

We anonymized interviewees and assigned them sequential numbers in the order they were interviewed. Each interview was conducted by two female research nurses, with LXJ leading the interview and DJY or SST responsible for taking notes. All team members had received training in qualitative research. Before the interview, the researcher explained the purpose, significance, and principle of voluntary participation in detail and obtained written informed consent. At the time of the formal interview, the following information was obtained through a questionnaire and electronic medical records: (a) patient demographic data, including age, gender, educational level, employment status, and financial situation; (b) disease-related information, covering nasopharyngeal carcinoma stage, treatment stage, disease status (comorbidity), nutritional status (PG-SGA), BMI, and albumin (the latest measurement). These data were used to analyze the associations between participants' nutritional management behaviors and their social background and disease status. All interviews were conducted in a quiet single room, and family members were allowed to accompany. The entire interview process was audio-recorded, during which contextual details and non-verbal cues were also captured. Each interview lasted approximately 25 to 40 min.

### 2.5 Data analysis

#### 2.5.1 Establishing patient persona label dimensions

Interview transcripts were transcribed verbatim within 24 h. The interview content and data were cross-checked and confirmed by both parties, and then returned to the participants for their comments. We utilized NVivo 15 software for data storage and qualitative data coding (25). We used directed content analysis with the three components of FBM (motivation, ability, and prompt) as our initial coding framework to detect nutritional self-management behavior characteristics. This approach was selected because it is particularly suited for studies aiming to validate or extend a theoretical framework, in this case the Fogg Behavior Model, while remaining open to emergent codes not fully captured by the pre-existing framework (26). The process is as follows: (1) Two researchers independently and repeatedly read through all interview transcripts to identify sentences and keywords closely related to nutritional self-management; (2) Marked paragraphs were classified with the initial coding framework when possible, and assigned new codes when the initial coding framework could not classify texts; (3) Semantically comparable encodings were integrated to form labels. Further consolidations of these labels into separate subcategories were made to create a hierarchical structure for labeling, so that patient characteristics can be systematically described.

#### 2.5.2 Manually extracting features to construct patient personas

Using the labeling system, the team extracted and synthesized features from each patient's interview data to generate initial personas. Participants then performed member checking to assess and revise their corresponding personas. In the grouping phase, patients were assigned to groups via pattern recognition based on similarities in motivation, ability, prompt, and related characteristics, maximizing within-group homogeneity and between-group heterogeneity. The team held multiple consensus discussions to refine classification logic, nomenclature, and alignment with the Fogg Behavior Model until thematic saturation. Each persona was structured to include group characteristics and core definitions, providing a theoretical basis for subsequent interventions.

#### 2.5.3 Statistical analysis

Data analysis in this study was performed using SPSS 31.0 software to investigate whether specific patient subgroups were significantly associated with poorer nutritional status. Continuous variables (BMI, albumin) were non-normally distributed and had small sample sizes; therefore, they were described using the median (P_25_, P_75_). Categorical variables (PG-SGA classification) were described using frequencies. The Kruskal-Wallis H-test was used to compare BMI and albumin levels across groups. In contrast, the Fisher-Freeman-Halton exact test was used to compare the distribution of PG-SGA classifications across groups. Based on clinical a priori hypotheses, exploratory *post-hoc* comparisons were further conducted between specific persona groups using the Mann-Whitney U-test. A two-sided *P* < 0.05 was considered statistically significant.

### 2.6 Rigor

This study adhered to the four criteria proposed by Lincoln and Guba to ensure research trustworthiness. Regarding credibility, triangulation was adopted: each interview was led by one researcher while another researcher performed persistent observation and recorded non-verbal cues. Preliminary results were returned to the participants for member checking to ensure that the findings authentically reflected their experiences. Regarding dependability, a clear audit trail was established, with comprehensive documentation of the study design, data collection, transcription, and analysis processes. Regarding confirmability, none of the researchers had any prior contact with the patients. During the interviews, active listening and open-ended questions were emphasized, allowing participants to express themselves freely and reducing the effect of social desirability bias. The research team maintained a neutral stance toward the data during the analysis phase. The sample size met the criterion of code saturation. Regarding transferability, we provided thick descriptions of the research context, participants, and study processes.

### 2.7 Ethical considerations

The Ethics Committee of Chongqing University Cancer Hospital approved this study (Approval No.: CZLL2025-135-001). All interviewees signed an informed consent form, and participants were allowed to withdraw from the study at any time. The interviewees' privacy and personal information were fully protected during the research process. All audio recordings and written records were for research purposes only.

## 3 Results

### 3.1 Demographic characteristics of participants

A total of 21 patients with nasopharyngeal carcinoma were enrolled in this study. The age ranged from 34 to 69 years, with a mean age of 50.10 ± 8.56 years. Among them, 12 were male, and 9 were female. Nutritionally, all patients exhibited albumin levels within the normal range (35.0–55.0 g/L) but presented with at least one NIS. Malnutrition was present in 61.9% of participants (PG-SGA > 1), comprising 33.33% with mild, 9.52% with moderate, and 19.05% with severe malnutrition. Individual characteristics are shown in Table 2.

**Table 2:** Individual characteristics (*n* = 21).

ID	Age	Gender	Education	Employment	Stage	Treatment	Treatment process	NIS	PG-SGA
P1	37	Female	Undergraduate	Employed	II	Chemoradiotherapy	9f	③④⑥	1
P2	55	Female	Middle school	Employed	III	Chemoradiotherapy	2f	①	1
P3	45	Man	High school	Unemployed	IVB	Chemotherapy	Maintenance treatment	①③⑥	6
P4	56	Female	Middle school	Retired	III	Chemoradiotherapy	23f	①②③⑥	11
P5	62	Man	High school	Retired	III	Chemoradiotherapy	22f	②③④⑤⑥	10
P6	57	Man	High school	Retired	IVA	Chemoradiotherapy	17f	②③④⑤⑥	9
P7	54	Man	Middle school	Unemployed	IVA	Immunotherapy	Maintenance treatment	⑥	2
P8	43	Female	Primary school	Unemployed	II	Chemoradiotherapy	27f	②③⑥	8
P9	51	Female	Primary school	Unemployed	IVA	Chemoradiotherapy	27f	①③④⑥	1
P10	43	Man	Primary school	Unemployed	IVB	Induction chemotherapy	Induction chemotherapy	①	1
P11	55	Man	Middle school	Unemployed	III	Chemoradiotherapy	9f	①④⑥	2
P12	43	Man	Undergraduate	Unemployed	III	Chemoradiotherapy	35f	①②③⑤	3
P13	69	Man	Middle school	Retired	III	Chemoradiotherapy	23f	②③④⑥	12
P14	40	Female	Middle school	Employed	III	Chemoradiotherapy	2f	②⑤	1
P15	45	Female	High school	Unemployed	II	Chemoradiotherapy	23f	①②③④⑤	1
P16	55	Female	Middle school	Employed	III	Chemoradiotherapy	13f	①②⑤	3
P17	53	Man	Primary school	Unemployed	III	Chemoradiotherapy	9f	①	1
P18	52	Man	Primary school	Unemployed	III	Chemoradiotherapy	31f	①②⑤⑥	2
P19	58	Man	Primary school	Retired	IVA	Chemoradiotherapy	17f	①②⑤⑥	2
P20	48	Female	Middle school	Employed	III	Induction chemotherapy	Induction chemotherapy	①	1
P21	34	Man	Middle school	Employed	III	Induction chemotherapy	Induction chemotherapy	①⑥	3

f, fraction; ①Nausea and vomiting; ②Radioactive stomatitis; ③Xerostomia; ④Hypogeusia; ⑤Dysphagia; ⑥Anorexia; PG-SGA: 0–1, well nourished; 2–3, mild malnutrition; 4–8, moderate malnutrition; ≥9, severe malnutrition.

### 3.2 Tailored personas for nutritional self-management in nasopharyngeal carcinoma patients

Based on interview findings, a total of 97 labels were extracted, covering motivations (17), abilities (39), prompts (10), family support (17), and psychological states (14). Based on five core dimensions (motivation, ability, prompt, psychological state, and family support), the personas for nutritional self-management among nasopharyngeal carcinoma patients were categorized into five types: (a) proactive managers (*n* = 4), symptom-driven active managers (*n* = 6), survival-driven passive copers (*n* = 6), cognition-behavior disconnected individuals (*n* = 3), and family-led passive compliers (*n* = 2). Detailed characteristics for each category are shown in Table 3. Thematic Saturation Grid and Diagram of the Theme Development Process; see Supplementary material.

**Table 3:** Tailored personas and intervention strategies for nutritional self-management for nasopharyngeal carcinoma patients.

Name	Proactive managers	Symptom-driven active managers	Survival-driven passive copers	Cognition-behavior disconnected individuals	Family-led passive compliers
Individual	P01-P02-P14-P20	P03-P06-P08-P10-P12-P16	P05-P07-P11-P13-P18-P19	P04-P15-P17	P09-P21
Age (year)	37/40/48/55	43/43/43/45/55/57	52/54/55/58/62/69	45/53/56	34/51
Gender	4 Female	4 Male + 2 Female	6 Male	1 Male + 2 Female	1 Male + 1 Female
Treatment process	Induction chemotherapy phase to the early radiotherapy phase	Mid-stage radiotherapy phase to maintenance treatment phase	Early radiotherapy phase to maintenance treatment phase	Early to mid-stage radiotherapy phase	Induction chemotherapy phase, late phase of radiotherapy
Education	Secondary school – Bachelor's Degree	Primary school – Bachelor's Degree	Primary school-Middle school	Primary school - High school	Primary school - Middle school
Motivation	• Proactive Prevention and Health Promotion	• Ensure the continuity of treatment, harm-avoidance	• Meet survival needs and alleviate symptoms	• Recognize the importance of nutritional management • There is a disconnect between cognition and behavior	• Patients exhibit low self-management motivation • Family members are the primary agents of nutritional management.
Ability	• High self-efficacy and resource integration ability • Possesses a clear dietary plan	• Good nutritional management capabilities, but subject to numerous objective constraints. • Possesses a fundamental dietary plan	• Lack of knowledge and skills, resulting in limited execution capabilities • No dietary plan, with a monotonous eating pattern	• Has a certain ability of self-management, but limited by personal preference and/or cognitive bias and/or physical symptoms. • During the hospital stay, there was no nutritional planning, with a preference instead for home-based recuperation.	• The patient's self-management skills are relatively weak. • Patient compliance is good • The family member possesses sound nutritional management capabilities.
Prompt	• External guidance, inner awareness of health and established health habits.	• Symptoms and medical events (such as treatment discontinuation)	• Severe nutrition impact symptoms	• Symptoms and inherent notions (such as, “green food” belief, vegetarianism)	• Family's proactive preparation, provision, supervision and encouragement
Mental state	• Positive mindset	• Anxiety and reflection coexist	• Negative mindset	• Optimistic mindset	• Coexisting psychological dependence and anxiety
Family support	• Strong family support with Professional nutritional support • Patient-lead	• Good family support with basic dietary care and emotional assistance • Patient-lead	• Limited family support with basic care and emotional companionship • Patient-lead	• Limited family support with basic care and emotional companionship • Patient-lead	• Adequate but Non-empowering Family Support • Family-lead
Intervention strategy	• Optimize prompts through resource alignment, earlier intervention, mobile health, and family-involved co-management.	• Provide targeted support to address barriers, including meal costs, limited hospital food accessibility, poor family support, and scheduling conflicts.	• Raise awareness of the importance of nutrition and simplify the operational complexity of nutritional management for patients.	• Enhance awareness of the importance of inpatient nutritional management while respecting dietary preferences to nudge behavioral change.	• Implement dyadic nutritional interventions for patients and their families

#### 3.2.1 Persona A: proactive managers

**Description**: This patient group consists mainly of middle-aged women aged between 37 and 55. From the onset of treatment, the patient exhibits a clear awareness of preventive nutritional management. The motivation behind this behavior lies in actively optimizing nutritional status to enhance treatment tolerance, improve prognosis and enhance quality of life, demonstrating a clear focus on personal benefit. This population exhibits robust self-management capabilities. Furthermore, they maintain a positive mindset and receive strong family support. Their nutritional management is sustained and maintained by multiple behavioral prompts.

**Key characteristics:**

- ***Clear motivation***: Centred on proactive prevention and holistic health promotion.
- ***Comprehensive ability***: Possess strong self-management capabilities, including formulating dietary plans, conducting regular self-monitoring, and proactively integrating multi-channel information resources.
- ***Diverse and internalized prompt****:* Behavior is not only externally guided but also stems from intrinsic awareness of health status and proactive maintenance of daily health habits.
- ***Positive mindset***: They can rationally regard nasopharyngeal carcinoma as a manageable chronic disease and face the reality proactively.
- ***Strong family support***: One or more family members have a healthcare background and thus can adequately and practically provide nutritional care.

**Quotes:**

**P02:** “*I want to build up my strength to get through the subsequent radiotherapy and chemotherapy. Nutrition is crucial—my sister, brother-in-law and daughter are all doctors, who give me dietary advice. I also like to look up relevant information on Baidu*.”

**P14:** “*Don't wait until symptoms appear to take intervention measures. I started adjusting my diet regularly as soon as I began treatment, hoping to maintain a good nutritional status. I keep a close eye on myself and monitor indicators like white blood cell count, platelet count and albumin levels. My diet consists of high-protein foods and vegetables, and is kept light and easily digestible*.”

#### 3.2.2 Persona B: symptom-driven active managers

**Description:** The age range of this population is 43 to 57 years, with a slightly higher proportion of males, and 66.7% have anemia and moderate malnutrition. In addition, 83% have Grade 1 to 2 radiation-induced oral mucositis. Their nutrition management behaviors are driven by adverse symptoms, aiming to alleviate discomfort and avoid treatment interruption, which reflects a distinct harm-avoidance characteristic. They have sound basic management capabilities and medication compliance and can proactively adjust their diets, seek professional support, or retrieve nutrition-related information independently according to their symptoms. However, this group experiences a certain degree of treatment efficacy anxiety. Family support, centered on basic dietary care and emotional assistance, fails to provide professional nutrition information.

**Key characteristics:**

- ***Clear motivation***: Ensure the continuity of treatment, with harm-avoidance as the primary goal;
- ***Capable yet constrained***: They have basic self-management competencies, can proactively adjust their diets and seek relevant information, but are susceptible to economic, environmental or cognitive constraints;
- ***Prompted by symptoms and medical events***: Behaviors are primarily prompted by negative events such as physical discomfort, abnormal indicators, treatment delays, and treatment interruptions.
- ***Anxiety and reflection coexist***: Concerned about recurrence and treatment outcomes, some patients can thus engage in positive reflection on nutrition management, which drives behavioral adjustments.
- ***Good family support***: Centered on basic dietary care and emotional assistance, yet lacking professional nutritional empowerment.

**Quotes:**

**P03:** “*I have a blood test every week. If any indicator is abnormal, I will adjust my diet to prioritize foods that target the problematic index. If my red blood cell count is low, I will buy spinach and pork liver. After chemotherapy, when my platelet count drops, I drink Wu Hong soup (a traditional Chinese medicine formula consisting of five red-colored ingredients) every day, which works well in combination with medication*.”

**P06**: “*When I had a sore throat, my wife would cook porridge specially for me, adding lean meat, carrots and green vegetables, and I forced myself to eat it. If you cannot eat, all your treatments will be affected. I was supposed to receive chemotherapy, but the schedule was delayed because of my low body weight and abnormal test indicators*.”

**P10**: “*The nutritional meal was a bit expensive, so I didn't order it. Since I live alone, I have to eat takeout, but I will choose foods that are better for my health. My family is not well-off, but we still buy nutritious foods. I am still young, and I have elderly parents and children to take care of. I have to keep going*.”

**P12**: “*To ensure I get enough nutrition, I force myself to eat as if it were a task. I used to love braised pork and barbecue. I wonder if those foods contributed to my illness—I will definitely pay more attention to my diet from now on. I will do more exercise after the treatment ends. Right now, I keep drinking milk, hoping to nourish and strengthen my body*.”

#### 3.2.3 Persona C: survival-driven passive copers

**Description:** This group consists mainly of middle-aged and older men aged between 52 and 69. 33.3% exhibited symptoms of severe malnutrition and anemia, whilst 66.7% suffered from grade 2–3 radiation-induced oral mucositis. Patients' motivation to eat is primarily to alleviate hunger and passively relieve symptoms. They exhibit poor self-management capabilities, lack the initiative to seek out nutritional information, and often harbor misconceptions about diet. Their daily diet is characterized by a casual approach of “eating whatever is available and making do,” with temporary adjustments to food texture only made when severe adverse reactions occur. Overall, they hold a negative mindset, coupled with a lack of family support.

**Key characteristics:**

- ***Weak motivation***: Dietary goals are limited to meeting basic survival needs and passively alleviatingsymptoms.
- ***Inadequate ability***: Poor self-management skills and a lack of initiative. Daily meals lack planning; the diet is monotonous and contains dietary misconceptions.
- ***Single Prompt***: Behavioral change is mainly prompted by severe nutrition impact symptoms, without sustained internal motivation or external prompts.
- ***Negative mindset***: Holding a negative view of nutritional management, regarding it as unrealistic or toofussy.
- ***Limited family support***: This tends to be confined to emotional support and basic care, making it difficult to provide substantive nutritional support.

**Quotes:**

**P05**: “*I do not usually pay attention to nutrition-related information, nor do I bother to research different dietary combinations. I want to eat according to my own preferences. My taste function has already been impaired, but I think nutrition management is of little use—the key lies in cancer treatment*.”

**P07**: “*Doctors did not make it clear to me what to eat for supplementation and what to avoid, so I have no idea at all! At present, my main problem is severe dental issues. My family tells me to cook food until it is tender. They also have no idea how to combine foods for proper nutrition. I am already quite old; I want to eat what I feel like eating*.”

**P13**: “*Anyway, I force myself to eat—first, to sustain my life; second, to try to prevent my health from deteriorating further. Regardless of whether the food is good or bad, nutritious or not, I force myself to eat a little as long as I can swallow it. I am a rural resident. As long as I can eat the food, it is fine. I never fuss over or pay attention to nutrition; I make do with whatever is available*.”

#### 3.2.4 Persona D: cognition-behavior disconnected individuals

**Description:** Patients in this category clearly recognize nutrition's importance for treatment. However, their dietary behaviors are still influenced by irrational factors such as ingrained beliefs, personal dietary preferences, and adverse symptoms, resulting in a disconnect between cognition and behavior. These dietary changes are largely reactive measures aimed at alleviating symptoms. The dietary structure during hospitalization is monotonous and accompanied by misconceptions (e.g., “homegrown rural produce is the most nutritious”). These patients postpone nutrition-improving behaviors until after discharge, lacking specific dietary plans during the critical inpatient period. Family support is limited to basic daily care, without effective nutritional guidance or supervision.

**Key characteristics:**

- ***Disconnect between cognition and behavior*:** Recognizing the importance of nutritional management, yet finding one's actions constrained by dietary preferences, misconceptions or physical symptoms.
- ***Passive coping-oriented self-management*:** A lack of systematic dietary planning; relying on a monotonous diet of vegetarian food, porridge and soups, with poor compliance.
- ***Prompts stem from symptoms and ingrained beliefs***: Behaviors are driven by physical discomfort, abnormal indicators, or ingrained perceptions of “green food.”
- ***Optimism with future-oriented delays*:** Hold an optimistic mindset but postpone nutritional improvements until discharge, neglecting active intervention in the critical inpatient period.
- ***Limited family support***: Restricted to emotional and daily care; fail to provide effective nutritional cognition and behavioral empowerment.

**Quotes:**

**P4**: “*Nutrition is important—otherwise, I wouldn't even be able to stand up... But my throat hurts so badly right now that I can only eat porridge and nothing else. I'll have to wait until I'm discharged to get proper nutrition.”*

**P15**: “*If my diet is irregular now, my platelet count will drop, my white blood cell count will also decrease, and all indicators will fail to meet the standards. Nutrient intake is crucial... But right now, I drink some soup casually and can't wait to be discharged so I can go home and recuperate. Homegrown food in the countryside is the most nutritious; everything bought in the city is junk food*.”

**P17**: “*Good nutrition keeps a person energetic. But I can't eat meat—it makes me feel nauseated... All the vegetables I eat at home are grown by myself without pesticides. I want to recover quickly and go back home, then take my time to nourish my body later*.”

#### 3.2.5 Persona E: family-led passive compliers

**Description**: In this category, family members act as the leaders and implementers of nutrition management, responsible for formulating and executing basic dietary plans. Patients themselves exhibit weak motivation and ability for self-management, and lack the willingness to actively acquire nutrition-related information, yet demonstrate good compliance with family arrangements. Their dietary behaviors are primarily prompted by external cues such as active preparation, supervision, and encouragement from family members. Anxiety and nervousness are prevalent among these patients, and the effectiveness and sustainability of their nutrition management are largely and directly dependent on the quality and level of family support.

**Key characteristics:**

- ***Reversal of roles***: Family members are the primary decision-makers, taking responsibility for the entire process, from meal planning to making adjustments.
- ***Ability dependence***: Patients have limited willingness and ability to self-manage, with dietary behaviors highly reliant on family arrangements.
- ***Externalized prompts***: Mainly dependent on family members' proactive preparation, daily supervision, and ongoing encouragement.
- ***Psychological dependence coexists with anxiety***: There is persistent worry and tension regarding the prognosis of the illness, coupled with a reliance on family support.
- ***Adequate but non-empowering family support***: Focuses on daily care and decision execution, yet does not improve patients' independent self-management capabilities.

**Quotes:**

**P09**: “*During my hospitalization, my son brings me meals. He says intravenous fluids alone can't achieve therapeutic effects; no nutrient solution can replace oral intake. He insists I drink pure milk every day. He prepares different types of meat for each meal, cooks them until tender for easy swallowing, and combines them with various fruits*.”

**P21**: “*When I had a good appetite a few days ago, my mother prepared three meals a day based on diabetic dietary guidelines—they lower blood sugar while being nutrient-dense and high in protein. However, my appetite became extremely poor after chemotherapy. When I can't eat, she encourages me to swallow food as if it were water...My mother has been buying sugar-free protein powder online for me because my blood sugar level is high..*.”

### 3.3 Theoretical mapping of the five personas in the Fogg Behavior Model

**Figure 1:**
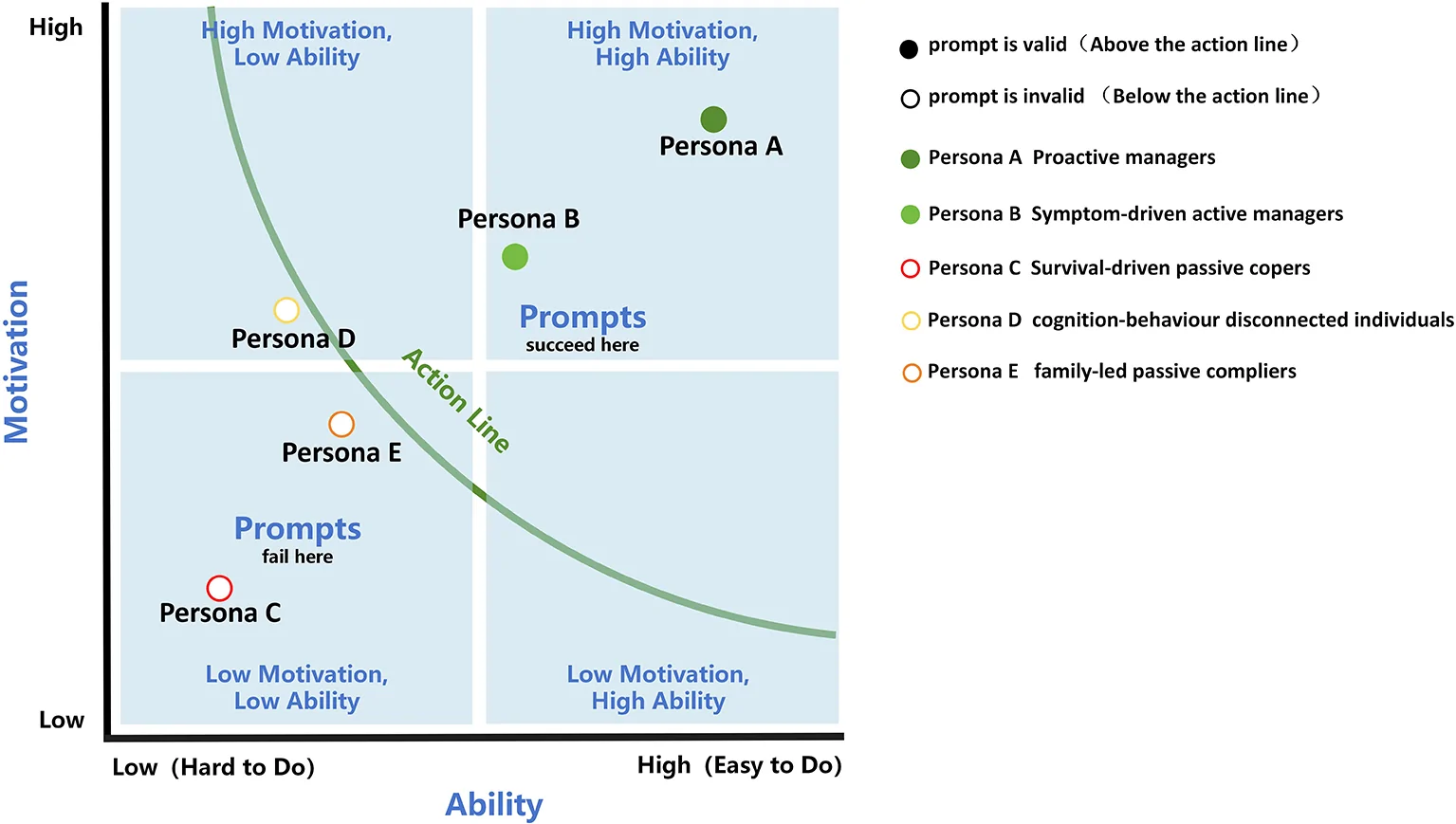
Theoretical mapping of patient personas onto the Fogg Behavior Model. Adapted with permission from the Fogg Behavior Model graphic, retrieved from https://www.behaviormodel.org. © Copyright 2026 by B.J. Fogg.

Based on the descriptive analysis, we mapped the five personas onto a 2 × 2 matrix derived from the Fogg Behavior Model (FBM), with Motivation (y-axis) defined as the willingness to perform normative nutritional behaviors, and Ability (x-axis) as the ease of executing these behaviors—shaped by time, cost, physical/cognitive effort, social norms, and routine disruption. The diagonal Action Line demarcates the threshold above which a prompt is more likely to trigger the target behavior, as shown in Figure 1.

- **Persona A-High Motivation, High Ability**. Positioned in the upper-right quadrant above the Action Line, reflecting that a simple prompt system can trigger the target behavior.
- **Persona B-High Motivation, Moderate Ability**. Located in the upper-right quadrant but close to the Action Line. This reflects a strong motivation to proactively adjust dietary habits to prevent symptoms from worsening and avoid delays in treatment. However, external obstacles such as financial constraints and hospital conditions limit the ability to do so.
- **Persona C-Low Motivation, Low Ability**. Situated in the lower-left quadrant, well below the Action Line, as they ignore the importance of nutritional management and lack knowledge and skills in nutrition management.
- **Persona D-High Motivation, Low Ability**. Located near the action line in the upper left quadrant, indicating recognition of nutritional importance; however, behaviors are influenced by inherent cognitive biases and dietary preferences, resulting in inadequate nutritional management.
- **Persona E-Moderate Motivation, Low Ability**. Located in the lower-left quadrant, near the bottom of the action line, the patient's initiative and ability regarding nutritional management are low. Still, family involvement can help compensate for some of these shortcomings.

### 3.4 Results of the statistical analysis of nutritional indicators

The distribution of nutritional indicators across the five personas is shown in Table 4. The Kruskal-Wallis H-test revealed no significant differences in BMI among the personas (*H* = 1.379, *P* = 0.848). The overall comparison of albumin levels across the five groups also failed to reach statistical significance (*H* = 5.063, *P* = 0.281); however, the rank-medians showed a clear gradient, with Persona A having the highest average albumin level (rank-median 16.50) and Persona C the lowest (rank-median 7.83). Based on clinical a priori hypotheses, exploratory *post-hoc* comparisons revealed that Persona C had a lower albumin mean rank than Persona A (Mann-Whitney *U* = 3.000, *P* = 0.055), approaching statistical significance.

**Table 4:** The nutritional indicators and statistical results for the five personas.

Persona	*N*	BMI (kg/m^2)^ Median (P25, P75)	Albumin (g/L) Median (P25, P75)	Mean rank of albumin	PG-SGAgrade (1/2/3/4), *n*
A	4	22.75 (22.04, 27.17)	47.90 (42.95, 49.48)	16.50	4/0/0/0
B	6	22.50 (17.01, 24.68)	45.00 (42.50, 46.10)	11.42	1/2/2/1
C	6	22.28 (19.57, 24.35)	40.80 (38.95, 44.60)	7.83	0/4/0/2
D	3	22.03 (21.48, −)	43.00 (42.20, −)	10.83	2/0/0/1
E	2	24.30 (21.49, −)	40.60 (34.90, −)	8.50	1/1/0/0
Overall test	21	*H* = 1.041, *P* = 0.904	*H* = 5.597, *P* = 0.231	-	Fisher-Freeman-Halton = 15.661, *P* = 0.061
*Post-hoc* exploratory (A vs. C)	10	-	*U* = 3.000, *P* = 0.050	-	-

BMI, body mass index; PG-SGA, Patient Generated Subjective Global Assessment; P_25_, 25th percentile; P_75_, 75th percentile. “-” indicates that SPSS could not compute the 75th percentile because of the small subsample size (*n* < 4*n* < 4). Between-group comparisons for continuous variables were performed using the Kruskal-Wallis H-test; the distribution of PG-SGA grades was assessed with the Fisher-Freeman-Halton exact test. The post-hoc comparison between Persona A and Persona C was conducted using the Mann-Whitney U-test.

Regarding subjective nutritional assessment, the Fisher-Freeman-Halton exact test showed a marginally significant difference in the distribution of PG-SGA grades among the five groups (*P* = 0.061). Cross-tabulation analysis indicated that all patients in Persona A (4/4) were classified as well-nourished (Grade 1). In contrast, patients with moderate to severe malnutrition (Grades 3 and 4) were primarily concentrated in Persona B (3/6) and Persona C (2/6). Based on a comprehensive analysis of subjective and objective indicators, as well as the textual descriptions of each persona, Persona B and C can be identified as the core high-risk groups for malnutrition and require special attention in clinical nutrition management.

## 4 Discussion

This study revealed heterogeneity in nutritional self-management among hospitalized patients with nasopharyngeal carcinoma and established five patient personas. These findings highlight the need for personalized interventions in NPC nutritional care, offering a theoretical basis and practical intervention targets for clinical application.

In this study, proactive managers were predominantly young and middle-aged women with relatively high levels of education. They showed strong health motivation, actively sought nutrition information, and maintained healthy dietary habits. This is consistent with previous findings, which have shown that female cancer survivors and individuals with higher educational attainment tend to exhibit greater nutritional literacy (27, 28). Moreover, most patients' family members work in healthcare, and they possess a deeper understanding of nutrition's role in recovery. They may, through their daily care, have gradually shaped the patients' health-management awareness and behavioral patterns. Therefore, for this high-ability, high-motivation population, optimizing prompts becomes the key to promoting nutritional management behaviors (21). The Regulatory Fit Theory suggests that individuals with a promotion focus who align with a beneficial information frame are more motivated to pursue their goals (29). Healthcare providers should tailor information framing to match patients' promotion focus, prioritizing benefit-oriented nutritional content. Intervention timing should align with their proactive prevention patterns, with systematic dietary education initiated at initial diagnosis and early conversion of awareness and behaviors promoted during induction chemotherapy (30). Delivery channels should accommodate their diverse information-seeking preferences, using mobile platforms for regular dissemination, interactive discussion forums, and progress tracking, while encouraging family involvement to establish a patient-family-provider collaborative network.

This study revealed that a considerable proportion of patients were symptom-driven active managers. The majority were diagnosed with malnutrition, accompanied by NIS such as anemia and oral mucositis. To avoid treatment delays due to symptom exacerbation, most patients proactively adjusted their dietary patterns (e.g., by modifying food types, textures, and flavors) to alleviate discomfort. As in previous studies, some patients attributed their disease to unhealthy lifestyle and dietary habits (31). The resultant regret and self-blame, though negative, concurrently motivated their intention to adopt healthier behaviors. Thus, providers should help patients objectively view dietary factors in disease development, avoid excessive self-blame, and offer evidence-based guidance for sustainable dietary regimens. Nevertheless, the nutritional management initiative of this population remained constrained by multiple barriers, including meal costs, limited hospital food accessibility, insufficient family support, and scheduling conflicts between radiotherapy and meal times, necessitating targeted supportive interventions. The “Food is Medicine” (FIM) initiative refers to a series of economically feasible food access pathways, including medically tailored meals (MTMs), medically tailored groceries (MTGs), food prescriptions, and food assistance (32). In 2025, Sinha et al. (33) proposed integrating precision nutrition with the FIM concept and underscored the immense potential of AI in its application. Therefore, hospitals could adopt this approach by leveraging AI to synthesize radiotherapy plans, financial status, preferences, and nutritional data into dynamic prescriptions and linking them with hospital or third-party meal services to deliver personalized, continuous nutritional support throughout treatment (34).

In this study, survival-driven passive copers were primarily middle-aged and older men with low educational levels. They viewed diet merely as a source of daily energy, paid more attention to cancer treatment than to nutrition, and consequently exhibited poor nutrition awareness and non-adherence to nutritional management during chemoradiotherapy. This aligned with the conceptual framework of health information avoidance among elderly patients with chronic diseases proposed by Lei et al. (35), who argued that cognitive conflict and low health literacy can easily prompt health information avoidance, leading to inadequate self-health management. In addition, patients in this group were troubled by symptoms accompanied by negative emotions such as tension and anxiety; these psychological states undermine their motivation and self-efficacy to maintain healthy nutritional behaviors. For these patients, nutritional intervention strategies should emphasize both reinforcing patients' recognition of the essential role of nutritional management and streamlining practical protocols (33). Nutrition and health information can be disseminated via social media apps that patients use daily (e.g., WeChat), and group chat features can be leveraged to conduct regular nutrition consultations, peer interaction, and incentive measures such as rewards (36). Furthermore, tailored culinary education or dietary prescriptions developed by dietitians or other specialists should be delivered via offline or online channels based on patients' symptom type, severity, dietary preferences, and access preferences, thereby enhancing the feasibility of nutritional management and promoting sustained adherence to healthy dietary behaviors.

This study found that cognition-behavior disconnected individuals highly recognized the importance of nutritional management, but their actual dietary behaviors deviated from clinical recommendations. In this group, many patients worked in agriculture. They held the ingrained belief that homegrown produce was nutritionally superior, leading them to rely on home dietary adjustments and neglect the critical window for inpatient nutritional intervention. A minority adhered to long-term vegetarianism, and their daily diets often fell short of clinical energy targets, further complicating nutritional management. This is consistent with Thaler's research findings, which indicate that decisions regarding health behaviors are often influenced by irrational factors such as cognitive biases and immediate preferences, and that simply conveying rational knowledge is insufficient to reverse behavioral inertia effectively (37). Based on this, the non-mandatory interventions advocated by the Nudge Theory, such as optimizing choice architecture, environmental design, and reinforcing positive information, offer a feasible pathway for nutritional interventions targeting this patient subgroup (37). Within hospital wards, promotional slogans and educational displays can be used to foster an atmosphere that emphasizes the importance of nutritional management during hospitalization (38). Hospital dining areas can use graphic nutrition labels to display ingredient sources and nutritional content, and position healthy meals prominently to reduce the cognitive effort to select suitable options (39). For vegetarian patients, nutritional gaps can be assessed and addressed with oral supplements or fortified foods, while respecting their dietary beliefs.

Among family-led passive compliers, patients had insufficient motivation and ability for nutritional self-management, with dietary decisions largely delegated to family members. This may stem from patients' lack of nutritional knowledge, treatment-related side effects that diminish eating enjoyment, and diagnosis-related anxiety that reinforces dependency, which contribute to passive acceptance rather than active engagement in dietary choices (40). In Chinese culture, the perceived responsibility to care for ill family members prompts relatives to take over caregiving tasks and proactively engage in patient-provider communication. Previous research has shown that family or caregiver involvement facilitates healthy dietary changes among cancer survivors (41). However, full decision-making authority may undermine patient autonomy in nutritional management, and prolonged caregiving burden and decision-making pressure may induce caregiver burnout and anxiety, thereby compromising nutrition care quality (42). Thus, nutritional interventions must address unilateral decision-making and adopt dyadic approaches that engage patients and families as a unit. A randomized controlled trial conducted by Molassiotis et al. (43) demonstrated that a family-centered psychosocial nutrition intervention, which focused on improving nutrition-related communication between dyads and setting shared nutrition goals, can alleviate eating difficulties and enhance eating enjoyment and quality of life. This suggests that nutrition management programs could be developed based on dyadic theory, incorporating dyadic nutrition tasks tied to daily anchor moments, such as before meals or before taking medication, to reduce the difficulty of implementation (44).

## 5 Limitation

First, the generalizability of our findings may be limited by the single-center design, as all participants were recruited from one Class A tertiary hospital located in Chongqing, China. Second, the interview data were collected and processed in Chinese, which may introduce translation bias when presenting the results in English. Furthermore, data saturation is relative. If the scope of data collection were significantly expanded, the interpretive dimensions and classification system could be further refined. At the same time, the findings of this study are were solely on the patient's perspective; future research could employ triangulation by incorporating other perspectives, such as those of caregivers or healthcare professionals. Finally, this study constructed user personas based on a single cross-sectional interview; future research could utilize longitudinal interviews further to capture dynamic changes in the nutritional management process.

## 6 Conclusion

This study employed a descriptive qualitative research methodology, utilizing the Fogg Behavior Model to reveal key differences in nutritional self-management among nasopharyngeal carcinoma patients across the three FBM components (motivation, ability, prompt) and inductive dimensions (psychological state, family support). Building upon this, we developed five representative user personas: proactive managers, symptom-driven active managers, survival-driven passive copers, cognition-behavior disconnected individuals, and family-led passive compliers. For each persona, this study proposes personalized intervention directions and strategy recommendations based on their key characteristics and behavioral logic in nutritional self-management, laying the groundwork for subsequent co-creation of interventions and quantitative validation.

## Data Availability

The original contributions presented in the study are included in the article/Supplementary material, further inquiries can be directed to the corresponding authors.

## References

[1] Chen Y Chan ATC Le Q Blanchard P Sun Y Ma J. Nasopharyngeal carcinoma. Lancet. (2019) 394:64–80. doi: 10.1016/S0140-6736(19)30956-0 31178151

[2] Chen B Zhan Z Xu Y Yu S Huang J Fang Y . Long-term trends in the burden of nasopharyngeal carcinoma in China: a comprehensive analysis from 1990 to 2021 and projections to 2030 based on the global burden of disease study 2021. Radiother Oncol. (2025) 202:110613. doi: 10.1016/j.radonc.2024.110613 39489428

[3] David G Sharon S Douglas A. NCCN guidelines for head and neck cancers, version 1.2026. (2025). Available online at: https://www.nccn.org/search-result?indexCatalogue=nccn-search-indexandsearchQuery=NCCN%20Guidelines%20Head%20and%20Neck%20Cancers (Accessed June 24, 2026).

[4] Miao J Wang L Hu C Lin S Tan SH Ong E . A multicenter prospective observational study of nutritional status on survival in locally advanced nasopharynx cancer treated by induction chemotherapy and chemoradiotherapy. J Clin Oncol. (2019) 37:6036. doi: 10.1200/JCO.2019.37.15_suppl.6036

[5] Shen L Chen C Li B Gao J Xia Y. High weight loss during radiation treatment changes the prognosis in under-/normal weight nasopharyngeal carcinoma patients for the worse: a retrospective analysis of 2433 cases. PLoS ONE. (2013) 8:e68660. doi: 10.1371/journal.pone.0068660 23869226 PMC3711826

[6] Wang P Lam Soh K Geok Soh K Xue L Ning C Tan Y . Systematic review of malnutrition risk factors to identify nutritionally at-risk patients with head and neck cancer. Clin J Oncol Nurs. (2024) 28:197–208. doi: 10.1188/24.CJON.197-208 38511915

[7] Wang P Huang X Liu Y Xue L Ning C Jiang L . Risk factors and the nomogram model for malnutrition in patients with nasopharyngeal carcinoma. Support Care Cancer. (2024) 32:256. doi: 10.1007/s00520-024-08459-6 38546900

[8] Li G Jiang X Qiu B Shen L Chen C Xia Y. Vicious circle of acute radiation toxicities and weight loss predicts poor prognosis for nasopharyngeal carcinoma patients receiving intensity modulated radiotherapy. J Cancer. (2017) 8:832–8. doi: 10.7150/jca.17458 28382146 PMC5381172

[9] Su L Lin Q Li R Hua Y Zhang H Song X . Prognostic value of nutritional impairment on treatment-related toxicity and survival in patients with nasopharyngeal carcinoma taking normal nutrition before radiotherapy. Head Neck. (2020) 42:3580–9. doi: 10.1002/hed.26426 32851765

[10] Tam V Ching J Yip S Kwong V Chan C Wong K . Examining patient-reported late toxicity and its association with quality of life and unmet need for symptom management among nasopharyngeal cancer survivors: a cross-sectional survey. Front Oncol. (2024) 14:1378973. doi: 10.3389/fonc.2024.1378973 38694788 PMC11061844

[11] Zhou Y Yang L Liang QT Ye XL Lu JM. Mediating effect of cancer-related fatigue between perceived social support and social alienation in survivors with nasopharyngeal cancer. Guangxi Med J. (2023) 45:816–20. doi: 10.11675/j.issn.0253-4304.2023.07.15. (in Chinese)

[12] Liu J Wang X Ye X Chen D. Improved health outcomes of nasopharyngeal carcinoma patients 3 years after treatment by the AI-assisted home enteral nutrition management. Front Nutr. (2025) 11:1481073. doi: 10.3389/fnut.2024.1481073 39839291 PMC11746109

[13] Zeng X Tang Y Zhan J Li Y Zou F. Nasogastric tube nutrition support enhances chemoradiotherapy compliance and alleviates mucosal reactions in nasopharyngeal carcinoma: a 856-case retrospective cohort study. Support Care Cancer. (2025) 33:854. doi: 10.1007/s00520-025-09915-7 40952517

[14] Xiao W Chan CWH Xiao J Wong CL Chow KM. Development of a nurse-led educational intervention program in managing the nutrition impact symptom cluster in patients with nasopharyngeal carcinoma following the medical research council framework. Asia Pac J Oncol Nurs. (2021) 8:653–61. doi: 10.4103/apjon.apjon-2141 34790849 PMC8522592

[15] Ji J Jiang D Xu Z Yang Y Qian K Zhang M. Continuous quality improvement of nutrition management during radiotherapy in patients with nasopharyngeal carcinoma. Nurs Open. (2021) 8:3261–70. doi: 10.1002/nop2.1039 34405584 PMC8510779

[16] Cheng Y Liu H Ma Y Yang T Hu W. Formulation of the best practice evidence-based nutrition management protocol for nasopharyngeal carcinoma patients undergoing chemoradiotherapy. Sichuan Univ (Med Sci). (2024) 55:1232–9. doi: 10.12182/20230860101. (in Chinese) 39507990 PMC11536255

[17] Xiao W Chan CW Jinnan X Lyu Q Gong N Wong CL . Managing the nutrition impact symptom cluster in patients with nasopharyngeal carcinoma using an educational intervention program: a pilot study. Eur J Oncol Nurs. (2021) 53:101980. doi: 10.1016/j.ejon.2021.101980 34275745

[18] Xia L Zhu XM Li SY Yang Y. Research progress in nutritional management of nasopharyngeal carcinoma patients in hospitals. J Chin Oncol. (2023) 29:1049–59. doi: 10.11735/j.issn.1671-170X.2023.12.B010 (in Chinese).

[19] Yang Z Xu L Gao Y Zhang C Wang A. Tailored personas for self-management in home-based cardiac rehabilitation for patients with coronary heart disease: a qualitative study. Int J Nurs Stud. (2025) 163:105000. doi: 10.1016/j.ijnurstu.2025.105000 39854909

[20] Ten Klooster I Wentzel J Sieverink F Linssen G Wesselink R van Gemert-Pijnen L. Personas for better targeted ehealth technologies: user-centered design approach. Jmir Hum Factors. (2022) 9:e24172. doi: 10.2196/24172 35289759 PMC8965674

[21] Fogg BJ. Tiny Habits: The Small Changes that Change Everything. Boston, MA: Houghton Mifflin Harcourt (2020).

[22] Agha S Tollefson D Paul S Green D Babigumira JB. Use of the Fogg behavior model to assess the impact of a social marketing campaign on condom use in Pakistan. J Health Commun. (2019) 24:284–92. doi: 10.1080/10810730.2019.1597952 30945612

[23] Alrige M Bitar H Meccawy M. Promoting precautionary behavior during the covid-19 pandemic: development and validation of a behavior-change messaging campaign. J Infect Public Health. (2021) 14:1727–32. doi: 10.1016/j.jiph.2021.09.026 34710783 PMC8491996

[24] Tong A Sainsbury P Craig J. Consolidated criteria for reporting qualitative research (coreq): a 32-item checklist for interviews and focus groups. Int J Qual Health Care. (2007) 19:349–57. doi: 10.1093/intqhc/mzm042 17872937

[25] Graneheim UH Lundman B. Qualitative content analysis in nursing research: concepts, procedures and measures to achieve trustworthiness. Nurse Educ Today. (2004) 24:105–12. doi: 10.1016/j.nedt.2003.10.001 14769454

[26] Assarroudi A Heshmati Nabavi F Armat MR Ebadi A Vaismoradi M. Directed qualitative content analysis: the description and elaboration of its underpinning methods and data analysis process. J Res Nurs. (2018) 23:42–55. doi: 10.1177/1744987117741667 34394406 PMC7932246

[27] Ryman C Warnicke C Hugosson S Zakrisson A Dahlberg K. Health literacy in cancer care: a systematic review. Eur J Oncol Nurs. (2024) 70:102582. doi: 10.1016/j.ejon.2024.102582 38608377

[28] Xia J Tang Z Yu J. An analysis of the current state of nutritional literacy among cancer survivors. Food Nutr China. (2024) 12:62–7. doi: 10.19870/j.cnki.11-3716/ts.2024.12.002. (in Chinese)

[29] Petrocchi S Ludolph R Labrie NHM Schulz P. Application of the theory of regulatory fit to promote adherence to evidence-based breast cancer screening recommendations: experimental versus longitudinal evidence. BMJ Open. (2020) 10:e37748. doi: 10.1136/bmjopen-2020-037748 33184078 PMC7662420

[30] Song ST Hu Q Lei XJ Du J Yan S Wang C. Research on journey map of oral health management in patients with nasopharyngeal carcinoma. J Nurses Train. (2026) 13:1370–7. doi: 10.16821/j.cnki.hsjx.2026.13.004. (in Chinese)

[31] Jiang J Yan M Fan Y Zhang J. Psychosocial adjustment experiences among nasopharyngeal carcinoma survivors: a qualitative study. Cancer Nurs. (2025) 48:E230–7. doi: 10.1097/NCC.0000000000001312 38032215

[32] Downer S Berkowitz SA Harlan TS Olstad DL Mozaffarian D. Food is medicine: actions to integrate food and nutrition into healthcare. BMJ. (2020) 369:m2482. doi: 10.1136/bmj.m2482 32601089 PMC7322667

[33] Sinha S Huey SL Shukla AP Kuriyan R Finkelstein JL Mehta S. Connecting precision nutrition with the food is medicine approach. Trends Endocrinol Metab. (2025) 36:511–20. doi: 10.1016/j.tem.2024.08.012 39341732

[34] Theodore Armand TP Nfor KA Kim J Kim H. Applications of artificial intelligence, machine learning, and deep learning in nutrition: a systematic review. Nutrients. (2024) 16:1073. doi: 10.3390/nu16071073 38613106 PMC11013624

[35] Lei YX Zhou YQ Li JN Meng LN. Concept analysis of health information avoidance in elderly patients with chronic diseases. Military Nurs. (2026) 43:38–42. doi: 10.3969/j.issn.2097-1826.2026.04.009. (in Chinese)

[36] Wang L Zou L Yi H Li T Zhou R Yang J . The implementation of online and offline hybrid weight management approach for pregnant women based on the Fogg behavior model in Hainan, China: a pilot randomized controlled trial. Bmc Pregnancy Childbirth. (2024) 24:516. doi: 10.1186/s12884-024-06699-2 39080659 PMC11289958

[37] Thaler RH. Misbehaving: The Making of Behavioral Economics. New York/London: W. W. Norton and Company (2015).

[38] Ledderer L Kjær M Madsen EK Busch J Fage-Butler A. Nudging in public health lifestyle interventions: a systematic literature review and metasynthesis. Health Educ Behav. (2020) 47:749–64. doi: 10.1177/1090198120931788 32517522

[39] Gao Z Li Z Zhuang X Ma G. Advantages of graphical nutrition facts label: faster attention capture and improved healthiness judgement. Ergonomics. (2023) 66:627–43. doi: 10.1080/00140139.2022.2107241 35894181

[40] Jiang J Fan Y Cheng Y Zhang J. Dyadic coping and factors associated with latent profiles among Chinese nasopharyngeal carcinoma patients and their spouses: a latent profile analysis. Eur J Oncol Nurs. (2026) 82:103195. doi: 10.1016/j.ejon.2026.103195 42030765

[41] Xu J Hoover RL Woodard N Leeman J Hirschey R. A systematic review of dietary interventions for cancer survivors and their families or caregivers. Nutrients. (2023) 16:56. doi: 10.3390/nu16010056 38201886 PMC10780967

[42] Fellia T Sarafis P Bouletis A Tzenetidis V Papathanasiou I Apostolidi T . Correlation of cancer caregiver's burden, stress, and their quality of life. Adv Exp Med Biol. (2023) 1425:267–73. doi: 10.1007/978-3-031-31986-0_25 37581800

[43] Molassiotis A Brown T Cheng HL Byrnes A Chan RJ Wyld D . The effects of a family-centered psychosocial-based nutrition intervention in patients with advanced cancer: the picnic2 pilot randomised controlled trial. Nutr J. (2021) 20:2. doi: 10.1186/s12937-020-00657-2 33388075 PMC7778804

[44] Duarte-Anselmi G Crane SM Ruiz MA Dintrans PV. Behavioral science meets public health: a scoping review of the Fogg behavior model in behavior change interventions. BMC Public Health. (2025) 25:3468. doi: 10.1186/s12889-025-24525-y 41088011 PMC12522219

