# Supplementary material for: Developing personas for nutritional self-management in nasopharyngeal carcinoma patients: a qualitative study based on the Fogg Behavior Model
Source: Front Nutr. 2026 Sep 1;13:1920095. doi: 10.3389/fnut.2026.1920095 (PMC13576166; doi:10.3389/fnut.2026.1920095)
Supplement: Supplementary file 1 [file Table_1.pdf]

Table S1 Thematic Saturation Grid

themes	Meaning of the Theme			
	Interview 1-6	Interview 7-12	Interview13-18	Interview after 19
Motivation	● Good nutritional status is the foundation of treatment and recovery（1）<br>● To sustain basic survival（5）<br>● Preventive Nutritional Management（2）<br>● Be in good physical condition to undergo further treatment（1）<br>● To Improve Blood Parameters（1）<br>● Preventing Recurrence and Striving for Long-Term Survival（3）<br>● To Enhance Immunity/Resistance（1）<br>● Good nutritional status is the foundation of life（4）<br>● Preference for oral intake over intravenous nutrition（4）	● To alleviate fatigue following chemotherapy（7）<br>● To gain weight（8）<br>● Driven by family responsibilities（10）	● To Strengthen the spleen and stomach（14）<br>● To improve mental state（17）<br>● Belief in the value of "green" foods（17）	● To Relieve xerostomia（19）<br>● To Control blood glucose（20）
Ability	● Strong willpower（1）<br>● Weak willpower（5）<br>● Good adherence to medical advice（1）<br>● Poor adherence to medical advice（5）<br>● Proactive selection of healthy foods（1）<br>● Modify dietary habits（1）<br>● Actively seek in-hospital professional resources（1）<br>● Modify food texture（1）	● Resistance to digital health tools（7）<br>● Time Resources（7）<br>● Dietary misconceptions（7）<br>● Lack of meal planning（7）<br>● Insufficient nutrition-related knowledge（7）<br>● Maintain nutrition within financial constraints（8）	● Integrate external advice with personal habits（14）<br>● Stimulate taste buds（14）<br>● Access to quality food resources: home-grown produce（17）	● Herbal tea for promoting salivation（19）<br>● Self-monitoring of blood glucose（20）

	● Acquire health information via online sources（1）<br>● Acceptance of digital health tools（1）<br>● Psychological resilience（1）<br>● Family support with medical background（1）<br>● Basic nutrition-related knowledge（1）<br>● Use of traditional Chinese herbal patches（1）<br>● Make preference-based choices within nutritional principles（1）<br>● Regular weight monitoring（2）<br>● Cooking skills（2）<br>● Regular physical exercise（2）<br>● Access to quality food resources: nutritional supplements（2）<br>● Regular intake of nutritional supplements and protein powder（2）<br>● Daily meal planning（3）<br>● Acquire health information through peer communication（3）<br>● Use of dietary therapy (e.g., "Five-Red Soup")（3）<br>● No active demand for nutritional information（3）<br>● Maintain limited intake despite treatment	● Regular intake of eggs and milk（11）		

	side effects（3）<br>● Flexible arrangement between supplements and regular diet（5）			
Prompt	● Health education from medical staff（1）<br>● Peer experience sharing（1）<br>● Send nutrition-related information（1）<br>● Physical symptoms（1）<br>● Abnormal blood parameters（1）<br>● Lifestyle habits（1）<br>● Supervision and Reminders from Family Members（2）<br>● Proactive Preparation by Caregivers（6）<br>● Negative experiences with previous treatments（6）	● Interruption or delay of treatment due to nutritional issues（8）		
Mental state	● Fear of the Unknown（1）<br>● Concerns about side effects（2）<br>● Fear of cancer recurrence（3）<br>● Optimistic Attitude（4）<br>● Belief that perseverance leads to success（4）<br>● Active cooperation with treatment（1）<br>● Avoid self-imposed psychological stress（1）	● Confusion about dietary choices（7）<br>● Perceived loneliness（8）<br>● Concerns About Treatment Outcomes（9）<br>● Anxiety due to loss of income（10）<br>● Worry about nutritional status（8）		● Confront reality with courage（20）<br>● View cancer as a chronic disease（20）
Family support	● Presence of a caregiver（1）<br>● Provision of daily living assistance（1）<br>● Family members with medical	● Absence of a caregiver（7）<br>● Children occupied with work or school（7）		● Primary caregiver: parent(s)（21）

	background（2）<br>● Family members with nutrition-related knowledge（2）<br>● Emotional support（1）<br>● Financial support （2）<br>● Disconnect between hospital and home care （3）<br>● Primary caregiver: spouse（1）<br>● Primary caregiver: adult children（2）<br>● Patient as decision-maker（1）	● Single-parent family（8）<br>● Carefully prepare three meals a day（9）<br>● Family members actively seek nutrition-related information（9）<br>● Family member as primary decision-maker（9）		
